# Supplementary material for: Impact of the Traditional Lecture Teaching Method and Dalcroze’s Body Rhythmic Teaching Method on the Teaching of Emotion in Music—A Cognitive Neuroscience Approach
Source: Brain Sci. 2025 Nov 21;15(12):1253. doi: 10.3390/brainsci15121253 (PMC12730861; doi:10.3390/brainsci15121253)
Supplement: Supplementary file 1 [file brainsci-15-01253-s001.zip › brainsci-3978668-supplementary.pdf]

S1 Questionnaire used in this study

Table S1 *Teaching quality evaluation scale，TS*

Guideline: Please rate the quality of the teacher's teaching by selecting the appropriate number from 1-5.

Level 1 indicator	Secondary indicators	Excellence	Good	Moderate	Acceptable	Poor
Teaching attitude	1. Well-prepared to teach, proficient in content, and in a positive mood.	5	4	3	2	1
Teaching content	2. Teaching content is systematic, substantial, and paced, progress arrangement is reasonable	5	4	3	2	1
	3. Teaching content is theoretically linked to practice and responds to the cutting edge of the discipline.	5	4	3	2	1
Teaching methods	4.To help students receive and understand relevant knowledge and develop their ability to think independently and creatively.	5	4	3	2	1
Teaching quality	5.Clearly organized, accurate in teaching knowledge, focused on points, and clarifies difficult points.	5	4	3	2	1
	6.Fluent expression, concise language, attractive strong.	5	4	3	2	1

S2 Musical materials used in this study

The Pipa is a quintessential plucked string instrument in traditional Chinese music, boasting a history spanning over two millennia. Its name traces back to the Qin Dynasty (221–206 BC), originating from a long-necked, round-bodied instrument. The term "Pipa" derived from the playing technique: "pi" referred to plucking the

strings forward, while "pa" meant hooking them backward. During the Northern and Southern Dynasties (420–589 AD), the crooked-neck Pipa from the Western Regions was introduced to Central China, merging with the indigenous straight-neck Pipa to form a new hybrid instrument. By the Tang Dynasty (618–907 AD), the Pipa reached its zenith, becoming widely popular in both court and folk music with refined playing techniques and standardized construction. Further improvements during the Ming (1368–1644) and Qing (1644–1912) dynasties culminated in its modern form—featuring six xiang (phase frets) and twenty-four pin (positional frets).

Contemporary Pipa performance adopts an upright posture, with the instrument held at approximately 45 degrees to the player's body (Fig. S1). The left hand presses the strings to control pitch, while the right hand—fitted with artificial nails—produces diverse timbres through techniques like plucking , lifting , tremolo, and strumming . Characterized by its bright, crystalline tone and distinctly articulated notes, the Pipa delivers crisp, resonant attacks with expressive tension. It masterfully conveys both delicate lyrical passages and grand dramatic scenes, solidifying its status as one of Chinese traditional music's most versatile and evocative instruments.

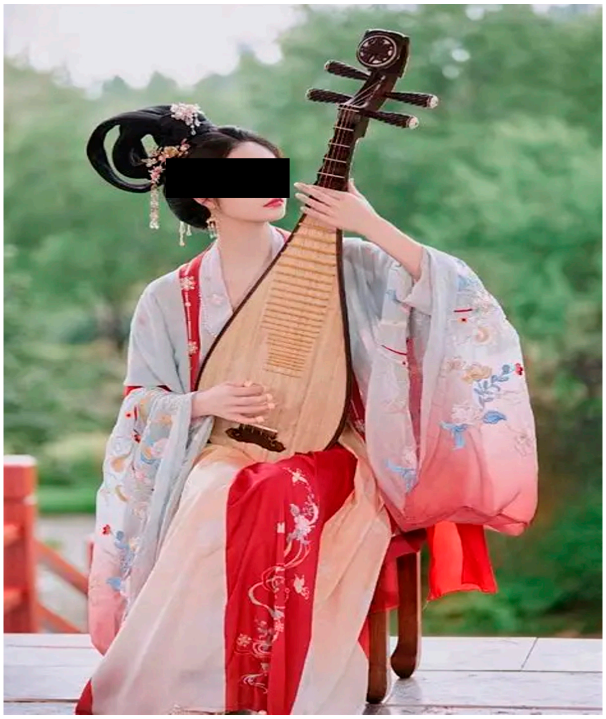

Figure S1 Examples of Pipa instrument performance

S2.1 *Titles of Pipa music pieces used in the experiment (samples)*

1.高山流水 (High mountain and flowing water)

2.送我一只玫瑰花 (Send me a rose)

3.渭水情 (The feeling of the Weishui River)

4.霸王卸甲 (The king unloads his armor)

S3 Teaching stage specific lesson plans

S3.1 *Traditional lecture* teaching *method*

Guideline:Welcome to the Music Appreciation class, next listen carefully to what mood the music is trying to express and what emotion it brings out in you.
**(High mountain and flowing water)**<br>Music 1, with a distinctive rhythm and smooth melody, is a board head tune with the local characteristics of Henan. According to the sheet music notation, the first section is cheerful and light, try to experience whether the music gives you this feeling. [Press 9 for music]<br>The second section is faster and lighter than the first according to the score,

try to feel it. [Press 9 for music]

In the third section, according to the notation, the fingering is swept, and the emotion is more intense, try to feel it. [Press 9 for music]

The last section repeats the first, and is also upbeat, so try to get a feel for it. [Press 9 for music]

(Send me a rose)

This music is a Xinjiang folk song with an upbeat and smooth melody. Try to experience if the music gives you this feeling. [Press 9 to hear the music]

The second section uses rotations and thicker intervals to play the main melody. The rhythmic accents are obvious, try to feel it. [Press 9 for music]

The third section is played with sweeping fingerings and a more intense mood. [Press 9 for music]

The last section repeats the first section, again with an upbeat feel. [press 9 for music]

(The feeling of the Weishui River)

This music combines the Northwest local theatre - Qinqiang, composed with the Northwest regional colours. Its emotion is delicate, melodious and soft. It expresses the feelings of longing for the homeland of Sanqin. Try to experience whether the music brings you this feeling. [Press 9 for music]

The second section continues the euphemistic and lyrical style of the first section, and uses the fingering method of pushing, pulling, chanting and kneading to reflect the local flavour. Try to feel it. [Press 9 for music]

The third section is based on the use of double wheels, euphemistic and deep, heartfelt and touching. Try to feel it. [Press 9 for music]

The fourth section adopts finger-swinging, expressing boldness and simplicity in the delicate and lingering. Try to feel it. [Press 9 for music]

(The king unloads his armor)

This music is taken from the Battle of Gaixia, a battle between Chu and Han, and the whole piece is sombre and melancholic. Try to experience whether the music gives you this feeling. [Press 9 to hear music]

The second section speeds up and is intense. It leads to the beginning of the war, try to feel it. [Press 9 for music]

The third section uses sweeping fingerings to bring the whole piece to its most intense atmosphere. It also signals the defeat of the war. Try to feel it. [press 9 for music]

The last section, the sad mood after the defeat of the war, and the previous part of the battle to form a sharp contrast, at this time the music wants to express the mood of grief and anger, grief. Try to feel it. [Press 9 for music]

S3.2 *Dalcroze's body rhythm teaching method*

Guideline:Welcome to Music Appreciation class, next listen carefully to the mood the music is trying to express and the emotions it brings out in you.

We will teach you to feel the music with your hands and arms, and to express it with body movements based on the changes you hear in rhythm, intensity and

speed. There is no right or wrong way to do these movements, so be bold with your body.
**(High mountain and flowing water)**<br>Music 1, we can tap the table with a single finger hand to find and imitate the rhythm of the first monotone. [Press 9 to hear the music]<br>For the second segment feel the change in tempo of the tapping with your fingers, you will notice the music speeding up. [Press 9 for music]<br>The third segment when you hear intense sounds we can use five fingers to make sounds together, pay attention to find the rhythm of the music and try to feel it. [Press 9 for music]<br>The last section of the music is thin with a single finger tapping, thick with five fingers and its, pay attention to adjust the fast and slow and the music of the beat in line. [Press 9 for music]
**(Send me a rose)**<br>This music focuses on imitating tone, rhythm and intensity. We can try to use the table as an African drum with both hands and beat the table together to the beat of the music. [Press 9 for music]<br>The second part of the rotation has a beautiful melody. Try to imitate the sound of the rotating fingers by tapping the table with your fingers on the tabletop. [Press 9 for music]<br>The third section of the sound weakened when the tapping becomes weaker, the back of the sound strong when the intensity becomes stronger, the sound of the most intense time, you can use both hands together tapping. [Press 9 for music]<br>For the last section, choose the action that you think is appropriate and feel it again. [Press 9 for music]
**(The feeling of the Weishui River)**<br>In this music we focus on feeling the melody, and you can choose a movement that you think expresses the melody. [Press 9 for music]<br>In the second section, if you don't have a suitable action, you can swing your arm in the air and change the amplitude of the swing according to the speed of the sound you hear to feel the delicate emotion the author wants to express. [Press 9 for music]<br>The third paragraph with the left hand swinging in the air at the same time, pay attention to the volume of change, weak time, the left hand swinging low amplitude, sound strong time amplitude become higher. [Press 9 for music]<br>The fourth section uses the method the teacher just taught you to feel the change of music again. [Press 9 for music]
**(The king unloads his armor)**<br>This music focuses on imitating sounds and rhythms, for example, we can make a muffled sound by hammering a table with one hand in a fist to the beat. [Press 9 for music]<br>Did you hear a loud, harsh sound in the second verse? We can mimic this squeaking sound by rubbing our fingernails back and forth against the table. [Press

9 for music]<br>The third section of the noisy sound is stronger, we can use both hands to rub the desktop to imitate the sound of the thick sense. [Press 9 for music]<br>The last section, use the method your teacher taught you to incorporate the music and listen to it again. [Press 9 for music]

S4 Supplementary Materials for Behavioral Outcomes

S4.1. Normality and Homogeneity of Variance Tests

The residual method was used for overall normality testing: after saving the standardized residuals from the mixed ANOVA, Shapiro-Wilk tests were conducted according to the teaching group variable. The results showed that the residuals generally followed a normal distribution (ps > .05), with only a few residuals slightly deviating from normality (p < .05). Since the absolute values of skewness and kurtosis were both less than 1, the Q-Q plot indicated that the data points were generally distributed along the diagonal line. Additionally, the Levene test for homogeneity of variances supported the homogeneity of variances across groups (ps > .05), meeting the requirements for parametric tests. Therefore, analysis of variance (ANOVA) was still employed.

S4.2 Analysis of the teaching effect of the three groups

Table S2. Detailed statistical data on subjects' information

	Instruction Strategy	$M \pm SD$	F	p	η_p^2
**Age**	Lecture teaching group	22.54 ± 2.47	0.94	0.394	0.018
	Body rhythm teaching group	23.03 ± 2.33			
	Control	22.24± 2.37			
**Gender**	Lecture teaching group	1.43 ± 0.5	1.15	0.321	0.022

	Body rhythm teaching group	1.43 ± 0.5			
	Control	1.27 ± 0.45			
**Fondness for Pipa music**	Lecture teaching group	3.71 ± 0.71	1.38	0.256	0.027
	Body rhythm teaching group	3.43 ± 0.78			
	Control	3.58 ± 0.66			
**Level of daily exposure to Pipa music**	Lecture teaching group	2.34 ± 0.91	0.009	0.992	0.00
	Body rhythm teaching group	2.31 ± 0.99			
	Control	2.33 ± 0.89			

Note. The results showed no significant group difference.

Table S3. The detailed statistic of pre-test performance

		Instruction Strategy	$M \pm SD$	F	p	η_p^2
**Familiarity with music**	Positive music	Lecture teaching group	2.64 ±1.36	0.007	0.993	0.00
		Body rhythm teaching group	2.61 ± 1.43			
		Control	2.65 ± 1.35			
	Negative music	Lecture teaching group	1.89 ± 1	0.439	0.646	0.009
		Body rhythm teaching group	2.06 ± 1.3			
		Control	2.14 ± 1.06			

**Music Emotion Recognition Valence**	Positive music	Lecture teaching group	5.23 ± 0.17	0.029	0.971	0.001
		Body rhythm teaching group	5.29 ± 0.17			
		Control	5.26 ± 0.17			
	Negative music	Lecture teaching group	3.26 ± 0.17	0.59	0.556	0.012
		Body rhythm teaching group	3 ± 0.17			
		Control	3.12 ± 0.17			
**Music Emotion Recognition Arousal**	Positive music	Lecture teaching group	4.89 ± 0.16	0.024	0.976	0.00
		Body rhythm teaching group	4.86 ± 0.16			
		Control	4.91 ± 0.17			
	Negative music	Lecture teaching group	4.63 ± 0.14	0.422	0.657	0.008
		Body rhythm teaching group	4.53 ± 0.14			
		Control	4.71 ± 0.14			
**Music Emotional Experience Valence**	Positive music	Lecture teaching group	5.06± 0.15	0.845	0.433	0.017
		Body rhythm teaching group	5.2 ± 0.15			

		Control	4.92 ± 0.15			
	Negative music	Lecture teaching group	3.4 ± 0.14	0.573	0.566	0.011
		Body rhythm teaching group	3.47 ± 0.14			
		Control	3.26 ± 0.15			
**Musical Emotional Experience Arousal**	Positive music	Lecture teaching group	5.19 ± 0.16	1.18	0.312	0.023
		Body rhythm teaching group	5.09 ± 0.16			
		Control	4.85 ± 0.16			
	Negative music	Lecture teaching group	4.67 ± 0.16	0.281	0.756	0.006
		Body rhythm teaching group	4.79 ± 0.16			
		Control	4.62 ± 0.16			

Note. The results showed no significant group difference.

S4.3 Supplementary Results of Subjective Scores for Music Emotional Processing

The results of a three-factor repeated measures ANOVA with 3 (teaching method: lecture teaching group, body rhythm teaching group, control group) × 2 (music valence: positive, negative) × 2 (time: pre-test, post-test) for subjective ratings of music emotion recognition (valence, arousal) and music emotion experience (valence, arousal) as the dependent variables were supplemented by the following results:

Regarding the valence of emotional recognition.The three-way interaction was significant, F (2, 100) = 19.27, $p < .001$, $\eta_p^2 = .28$. Using Bonferroni correction for simple simple effects analysis. For positive music emotional recognition valence: The lecture group's post-test (5.83 ± .16) was significantly higher than its pre-test (5.23 ± .17; $p < .001$); The body rhythm group's post-test (5.86 ± .16) was significantly higher than its pre-test (5.29 ± .17; $p < .001$); The control group's post-test (5 ± .16) was significantly lower than its pre-test (5.26 ± .17; $p = .044$). For negative music emotional recognition valence: The lecture group's post-test (2.76 ± .15) was significantly lower than its pre-test (3.26 ± .17; $p = .001$); The body rhythm group's post-test (2.31 ± .15) was significantly lower than its pre-test (3 ± .17; $p < .001$); The difference between the control group's post-test (3.29 ± .16) and pre-test (3.12 ± .17) was not significant ($p = .285$). Furthermore, the interaction between Music and Teaching Method was significant, F (2, 100) = 8.47, $p < .001$, $\eta_p^2 = .15$. Simple effects analysis showed, For positive music emotional recognition valence, the differences among the lecture group (5.53 ± .15), the body rhythm group (5.57 ± .15) and the control group (5.13 ± .15) were not significant ($p = .08$). For negative music emotional recognition valence, The difference between the lecture group (3 ± .14) and the control group (3.21 ± .15) was not significant ($p = .1$); The difference between the lecture group and the body rhythm group was not significant ($p = .25$); The body rhythm group (2.66 ± .14) was significantly lower than the control group ($p = .025$). Results for emotional recognition valence within groups showed, The positive music valence of the Lecture group (5.53 ± .15) was significantly higher than negative music

valence (3 ± .14; $p < .001$).The positive music valence of the Body rhythm group (5.57 ±.15) was significantly higher than negative music valence (2.66 ± .14; $p < .001$). The positive music valence of the Control group (5.13 ± .15) was significantly higher than negative music valence (3.21 ± .15; $p < .001$). Additionally, the interaction between Music and Time was significant, F (2, 100) = 28.36, $p < .001$, $\eta_p^2 = .22$. Simple effects analysis showed, The post-test score for positive music emotional recognition valence (5.56 ± .09) was significantly higher than the pre-test (5.26 ± 0.1; $p < .001$); The post-test score for negative music emotional recognition valence (2.79 ± .09) was significantly lower than the pre-test (3.13 ± 0.1; $p < .001$). Results for emotional recognition valence within Time points showed, The positive music valence of pre-test (5.26 ± 0.1) was significantly higher than negative music valence (3.13 ± 0.1; $p < .001$). The positive music valence of post-test (5.56 ± .09) was significantly higher than negative music valence (2.79 ± .09; $p < .001$). Finally, the main effect of Music was significant, F (2, 100) = 623.51, $p < .001$, $\eta_p^2 = .86$. The emotional recognition valence for positive music (5.41 ± .09) was significantly higher than for negative music (2.96 ± .08; $p < .001$).

Regarding emotional recognition arousal. The interaction between Time and Teaching Method was significant, F (2, 100) = 6.42, $p = .002$, $\eta_p^2 = .11$. Simple effects analysis revealed, The post-test score for the lecture group (5.01 ± .11) was significantly higher than its pre-test score (4.78 ± 0.12; $p = .018$). The post-test score for the body rhythm group (5.17 ± 0.11) was significantly higher than its pre-test score (4.69 ± .12; $p < .001$). The difference between the post-test (4.74 ± .12) and

pre-test (4.81 ± .13) for the control group was not significant (p = .537). Furthermore, the main effect of Music was significant, F (2, 100) = 6.69, p = .112, η_p^2 = .06. The emotional recognition arousal for positive music (4.98 ± .08) was significantly higher than for negative music (4.75 ± .07; p = .011). Finally, the main effect of Time was significant, F (2, 100) = 12.77, p = .001, η_p^2 = .11. The emotional recognition arousal at post-test (4.98 ± .07) was significantly higher than at pre-test (4.75 ± .07; p = .001).

Regarding emotional experience valence. The three-way interaction was significant, F (2, 100) = 9.67, p < .001, η_p^2 = .16. Using Bonferroni correction for simple simple effects analysis. For positive music emotional experience valence, The lecture group's post-test (5.47 ± .15) was significantly higher than its pre-test (5.06 ± .15; p = .001); The body rhythm group's post-test (5.84 ± .15) was significantly higher than its pre-test (5.2 ± .15; p < .001); The difference between the control group's post-test (4.91 ± .15) and pre-test (4.92 ± .15) was not significant (p = .907). For negative music emotional experience valence, The difference between the lecture group's post-test (3.19 ± .16) and pre-test (3.40 ± 0.14) was not significant (p = .16); The body rhythm group's post-test (2.79 ± .16) was significantly lower than its pre-test (3.47 ± 0.14; p < .001); The difference between the control group's post-test (3.21 ± .17) and pre-test (3.26 ± .15) was not significant (p = .771). Furthermore, the interaction between Music and Teaching Method was significant, F (2, 100) = 4.98, p = .009, η_p^2 = .09. Simple effects analysis showed. For positive music emotional experience valence, The difference between the lecture group (5.26 ± .13) and the control group (4.91± .14) was not significant (p = .208); The difference between the

lecture group and the body rhythm group was not significant (p = .513); The body rhythm group (5.52 ±.13) was significantly higher than the control group (p = .006). For negative music emotional experience valence, the differences among the lecture group (3.29 ± .13), the body rhythm group (3.13 ± .13) and the control group (3.24 ± .14) were not significant (p = .673). Results for emotional experience valence within groups showed, The positive music valence of lecture group (5.26 ± .13) was significantly higher than negative music valence (3.29 ± .13; $p < .001$); The positive music valence of body rhythm group(5.52 ±.13) was significantly higher than negative music valence (3.13 ±.13; $p < .001$); The positive music valence of control group(4.92 ± .14) was significantly higher than negative music valence (3.24 ±.14; $p < .001$). Additionally, the interaction between Music and Time was significant, F (2, 100) = 30.4, $p < .001$, $\eta_p^2 = .23$. Simple effects analysis showed: The post-test score for positive music emotional experience valence (5.41 ± .08) was significantly higher than the pre-test (5.06 ± .09; $p < .001$); The post-test score for negative music emotional experience valence (3.06 ± .10) was significantly lower than the pre-test score (3.38 ± .08; p = .001). Results for emotional experience valence within Time points showed, The positive music valence of pre-test(5.06 ± .09) was significantly higher than negative music valence (3.38 ± .08; $p < .001$); The positive music valence of post-test(5.41 ± .08) was significantly higher than negative music valence (3.06 ± .10; $p < .001$). Finally, the main effect of Music was significant, F (2, 100) = 477.53, $p < .001$, $\eta_p^2 = .83$. The emotional experience valence for positive music (5.23 ± .08) was significantly higher than for negative music (3.22 ± .08; $p < .001$).

Regarding emotional experience arousal. The interaction between Time and Teaching Method was significant, F (2, 100) = 8.57, $p < .001$, $\eta_p^2 = .15$. Simple effects analysis revealed. The post-test score for the lecture group (5.19 ± .13) was significantly higher than its pre-test (4.93 ± .12; $p = .012$); The post-test score for the body rhythm group (5.64 ± .13) was significantly higher than its pre-test (4.94 ± .12; $p < .001$); The difference between the post-test (4.89 ± .13) and pre-test (4.74 ± .13) for the control group was not significant ($p = .144$). Furthermore, the main effect of Music was significant, F (2, 100) = 12.91, $p = .001$, $\eta_p^2 = .11$.The emotional experience arousal for positive music (5.23 ± .08) was significantly higher than for negative music (4.88 ± .09; $p = .001$). Finally, the main effect of Time was significant, F (2, 100) = 40.69, $p < .001$, $\eta_p^2 = .29$. The emotional experience arousal at post-test (5.24 ± .08) was significantly higher than at pre-test (4.87 ± .07; $p < .001$).

S4.4. Supplementary information on the subjective scoring results of teaching quality evaluation

The evaluation of teaching quality includes four components: teachers' teaching attitudes, teaching methods, teaching qualities, and teaching content. Repeated measures analysis of variance (ANOVA) was conducted on the scores for each of these four components, specifically for teaching methods × music type. In the evaluation of teaching attitudes, the main effect of teaching methods was significant, with a significant difference between the two groups: F (1,68) = 12.55, $p = .001$, η_p^2= 0.16. The body rhythm teaching group (4.75 ± .1) was significantly better than the lecture teaching group (4.26 ± .1). In terms of teaching method scores, the two-factor

interaction was significant, F (1,68) = 12.65, p = .001, η_p^2 = .16. Simple effect analysis showed that when teaching positive music, the body rhythm teaching group (4.53 ± .12) was significantly higher than the lecture teaching group (3.7 ± .12; p < 0.001). When teaching negative music, the difference between the body rhythm teaching group (4.3 ± .13) and the lecture teaching group (4.01 ± .143) did not reach statistical significance (p = .12). In terms of teaching quality, the main effect of teaching method was significant, with a significant difference between the two groups, F (1,68) = 9.88, p = .002, η_p^2 = 0.13. The body rhythm teaching group (8.85 ± .18) was significantly better than the lecture teaching group (8.04 ± .18). In terms of teaching content evaluation, the difference between the two groups did not reach statistical significance (p = 0.16).

S5. fNIRS data acquisition

S5.1. Recording and Channel Layout of fNIRS

In this study, we used a NIRScout16-24 benchtop near-infrared functional brain imaging system (NIRx Medizintechnik GmbH, Germany) with a sampling rate of 7.8125Hz to monitor the cerebral cortex oxygenated hemoglobin (HbO) and deoxyhemoglobin (HbR) concentration changes. Given that the prefrontal cortex is closely associated with emotional processing (van Holstein & Floresco, 2020), and the right temporoparietal joint area is an important region for social interaction learning (Zillekens et al., 2019; Pan et al., 2020), the main brain regions observed in this study were prefrontal and the right temporoparietal joint area. We applied 8 emitters and 12 detectors to form 26 channels for each learner-teacher dichotomy (see Fig. 1). The

two types of optical poles were spaced apart with an adjacent spacing of 3 cm, the middle column of probes was aligned along the sagittal reference plane according to the international 10-20 system, and the central optical node of the lowest probe row was placed at Fpz. Four emitters and seven detectors were placed in the prefrontal cortex, forming 14 channels, and other optical probe sets were located in the right TPJ (4 emitters and 5 detectors, forming 11 channels). All probe sets were checked and adjusted to ensure consistency for each participant. Channel localization data were collected by a 3D virtual localization system (Polhemus, ColChester, VT, USA), and channel MNI coordinates were computed using the NIRS-SPM spatial alignment (Spatial Registration) function (See Table S4).

Table S4. ***Spatial Localization of fNIRS Channels***

Chanels	MNI coordinates			Broodmann's areas	Percentage of ooverlap
	x	y	z		
CH01	-12	73	-4	11 - Left Orbitofrontal area	0.5
CH02	2	68	13	10 - Left Frontopolar area	1
CH03	14	73	-4	11 - Right Orbitofrontal area	0.52
CH04	-18	72	10	10 - Left Frontopolar area	0.98
CH05	-25	68	15	10 - Left Frontopolar area	0.95
CH06	-8	66	28	10 - Left Frontopolar area	0.94
CH07	-16	58	38	9 - Left Dorsolateral prefrontal cortex	0.78
CH08	11	68	27	10 - Right Frontopolar area	0.97
CH09	21	72	10	10 - Right Frontopolar area	0.98
CH10	20	58	38	9 - Right Dorsolateral prefrontal cortex	0.83
CH11	28	68	16	10 - Right Frontopolar area	0.96
CH12	2	55	40	9 - Left Dorsolateral prefrontal cortex	0.91
CH13	-10	48	51	9 - Left Dorsolateral prefrontal cortex	0.84
CH14	13	48	52	9 - Right Dorsolateral prefrontal cortex	0.84
CH15	66	-19	44	1 - Right Primary Somatosensory Cortex	0.55
CH16	73	-23	7	22 - Right Superior Temporal Gyrus	0.72
CH17	70	-37	28	40 - Right Supramarginal gyrus part of Wernicke's area	0.4
CH18	57	-35	57	40 - Right Supramarginal gyrus part of Wernicke's area	0.5

CH19	61	-52	44	40 - Right Supramarginal gyrus part of Wernicke's area	0.75
CH20	49	-65	53	39 - Right Angular gyrus, part of Wernicke's area	0.68
CH21	72	-39	-10	20 - Right Inferior Temporal gyrus	0.52
CH22	68	-53	9	21 - Right Middle Temporal gyrus	0.4
CH23	62	-64	-6	37 - Right Fusiform gyrus	1
CH24	59	-66	26	39 - Right Angular gyrus, part of Wernicke's area	0.83
CH25	47	-78	36	39 - Right Angular gyrus, part of Wernicke's area	0.67
CH26	53	-79	10	19 - Right V3	0.76

Note: Some NIR observation channels may cover multiple brain regions, and this table only lists brain regions with a coincidence degree greater than 0.4.

S5.2 Brain Results Supplement

S5.2.1 Single Brain Activation Results Complementary

Table S5. ***Means and standard deviations of all channels of HBO in the three groups （N=103）***

Variables	Lecture teaching group (*n* = 35)				Body rhythms teaching group（*n* = 35）				Control group （*n* = 33）			
	Pre-test		Post-test		Pre-test		Post-test		Pre-test		Post-test	
	Positive Music	Negative Music	Positive Music	Negative Music	Positive Music	Negative Music	Positive Music	Negative Music	Positive Music	Negative Music	Positive Music	Negative Music
	M±SD	*M±SD*	*M±SD*	*M±SD*	*M±SD*	*M±SD*	*M±SD*	*M±SD*	*M±SD*	*M±SD*	*M±SD*	*M±SD*
lFPC	-0.018±0.006	-0.009±0.005	-0.009±0.005	0.009±0.05	-0.016±0.006	0.009±0.005	0.017±0.05	0.023±0.005	-0.001±0.006	0.001±0.005	-0.01±0.006	-0.004±0.006
rFPC	-0.019±0.007	-0.005±0.006	-0.012±0.006	0.004±0.005	-0.021±0.007	0.006±0.006	0.018±0.006	0.02±0.005	0.005±0.008	0.004±0.006	-0.004±0.007	-0.0003±0.005
lOFC	-0.026±0.009	-0.013±0.007	-0.02±0.009	0.006±0.007	-0.012±0.009	0.009±0.007	0.028±0.009	0.021±0.007	-0.009±0.009	0.007±0.008	-0.01±0.009	0.0005±0.007
rOFC	-0.006±0.01	-0.0001±0.008	-0.007±0.008	0.011±0.007	-0.012±0.01	0.012±0.008	0.024±0.008	0.022±0.007	0.00003±0.01	0.003±0.008	-0.001±0.008	0.004±0.007
ldlPFC	-0.022±0.009	-0.012±0.007	-0.016±0.011	0.01±0.006	-0.07±0.009	0.017±0.007	0.033±0.011	0.026±0.006	-0.003±0.009	-0.003±0.007	-0.016±0.011	-0.003±0.006
rdlPFC	-0.012±0.005	-0.004±0.006	-0.012±0.005	0.009±0.003	-0.016±0.005	0.006±0.006	0.011±0.005	0.01±0.003	-0.006±0.005	0.003±0.006	-0.005±0.005	0.001±0.003
rSI	0.015±0.026	0.015±0.015	0.051±0.033	0.012±0.015	0.049±0.026	0.041±0.015	0.074±0.033	0.037±0.015	-0.009±0.027	-0.005±0.015	-0.012±0.034	0.003±0.015
rANG	-0.011±0.007	-0.007±0.005	-0.008±0.009	-0.001±0.01	-0.002±0.007	-0.003±0.005	0.017±0.009	0.02±0.01	-0.003±0.007	-0.006±0.005	-0.009±0.009	0.006±0.01
rSMG	-0.009±0.006	-0.002±0.005	-0.005±0.005	-0.005±0.008	-0.007±0.006	0.002±0.005	0.005±0.005	0.006±0.008	0.004±0.006	-0.001±0.005	-0.004±0.006	0.003±0.008
rITG	-0.01±0.01	0.013±0.01	-0.009±0.011	0.003±0.007	-0.007±0.01	-0.014±0.01	0.011±0.011	-0.001±0.007	-0.016±0.01	-0.006±0.01	-0.002±0.012	0.0005±0.007

rTPG	-0.013±0.006	0.003±0.005	-0.01±0.005	-0.004±0.007	-0.005±0.006	-0.003±0.005	0.004±0.005	0.006±0.007	-0.008±0.007	-0.002±0.005	-0.005±0.005	0.002±0.007
rFFG	-0.014±0.008	-0.02±0.008	-0.002±0.008	-0.015±0.008	-0.008±0.008	0.001±0.008	0.002±0.008	0.014±0.008	0.001±0.009	-0.003±0.009	-0.003±0.008	-0.0037±0.009
rV3	-0.03±0.01	-0.001±0.009	-0.00002±0.008	-0.004±0.009	0.002±0.01	-0.001±0.009	0.003±0.008	0.02±0.009	-0.003±0.01	0.008±0.009	-0.015±0.009	0.0004±0.009
rSTG	-0.016±0.01	-0.002±0.012	0.005±0.01	0.009±0.008	-0.002±0.01	-0.014±0.012	0.007±0.01	0.014±0.008	-0.011±0.011	-0.006±0.012	-0.006±0.01	-0.001±0.008

A three-way repeated measures ANOVA with 3 (teaching method: lecture teaching group, body rhythm teaching group, control group) × 2 (music valence: positive, negative) × 2 (time: pre-test, post-test) was performed on the HBO means of the ROIs for the three groups. The results of multiple comparisons were corrected with FDR, and the supplementary results are shown below:

In the lFPC activation, the main effect of music type was significant, $F(1, 100) = 13.48$, $p_{FDR} < 0.001$, $\eta_p^2 = 0.12$, with negative music (.005 ± .002) HBO activation significantly higher than positive music (-.006 ± .002; $p < 0.001$). In the rFPC activation, the main effect of music type was significant, $F(1, 100) = 10.22$, $p_{FDR} = 0.011$, $\eta_p^2 = 0.09$, with negative music (.005 ± .002) HBO activation significantly higher than positive music (-.006 ± .003; $p = 0.002$). In lOFC activation, the main effect of music type was significant, $F(1, 100) = 10.22$, $p_{FDR} = 0.011$, $\eta_p^2 = 0.09$, with negative music (.005 ± .003) HBO activation significantly higher than positive music (-.008 ± .004; $p = 0.003$). In ldlPFC activation, the main effect of music type was significant, $F(1, 100) = 6.63$, $p_{FDR} = .026$, $\eta_p^2 = 0.06$, with negative music (.006 ± .003) HBO activation significantly higher than positive music (-.005 ± .005; $p = 0.011$). In the rdlPFC activation, the main effect of music type was significant, F(1, 100) = 16.02, $p_{FDR} < .001$, $\eta_p^2 = 0.14$, with negative music (.004 ± .002) HBO activation significantly higher than positive music (.007 ± .002; $p < .001$). In the rV3 activation, the main effect of music type was significant, $F(1, 100) = 8.27$, $p_{FDR} = .014$, $\eta_p^2 = 0.08$.

The activation of HBO in negative music (.004 ± .003) was significantly higher than that in positive music (-.007 ± .004; p = 0.005), See Figure S2a..

Additionally, in the activation of lFPC, the main effect of time was significant, $F(1, 100) = 16.96$, $p_{FDR} < .001$, $\eta_p^2 = 0.15$, with the post-test (.004 ± .002) HBO activation significantly higher than the pre-test (-.006 ± .002; $p < 0.001$). In the activation of rFPC, the main effect of time was significant, $F(1, 100) = 6.96$, $p_{FDR} = .007$, $\eta_p^2 = 0.07$, with post-test (.004 ± .002) HBO activation significantly higher than pre-test (–.005 ± .003; $p = 0.01$). In the activation of the lOFC, the main effect of time was significant, $F(1, 100) = 7.64$, $p_{FDR} = .025$, $\eta_p^2 = 0.07$, with post-test (.004 ± .003) HBO activation significantly higher than pre-test (–.007 ± .004; $p = .007$). In terms of activation in the ldlPFC, the main effect of time was significant, $F(1, 100) = 6.21$, $p_{FDR} = 0.039$, $\eta_p^2 = 0.06$, with post-test (.006 ± .004) HBO activation significantly higher than pre-test (–.005 ± .004; $p = .014$). In the activation of the rdlPFC, the main effect of time was significant, $F(1, 100) = 8.09$, $p_{FDR} = .023$, $\eta_p^2 = 0.08$. The activation of HBO in the post-test (.002 ± .002) was significantly higher than that in the pre-test (-.005 ± .002; $p = .005$), See Figure S2b.

Finally, in lFPC activation, the main effect of teaching method was significant, $F(2, 100) = 6.85$, $p_{FDR} = .009$, $\eta_p^2 = 0.12$. Pairwise comparisons showed that the difference in HBO activation between the lecture teaching group (-.007 ± .003) and the control group (-.003 ± .003) was not significant (p =.438). The body rhythm teaching group (.008 ± .003) had significantly higher HBO activation than the control

group (p =.008) and the lecture teaching group (p =.001). In rFPC activation, the main effect of teaching method was significant, $F(2, 100) = 4.23$, $p_{FDR} = 0.014$, $\eta_p^2 = 0.08$. Pairwise comparisons showed that the difference in HBO activation between the lecture teaching group (-.008 ± .003) and the control group (.001 ± .003) was not significant (p =.061). The body rhythm teaching group (.006 ± .003) had significantly higher HBO activation than the lecture teaching group (p =.005). There was no significant difference between the body rhythm teaching group and the control group (p =.359). In lOFC activation, the main effect of teaching method was significant, $F(2, 100) = 6.91$, $p_{FDR} = .009$, $\eta_p^2 = 0.12$. Pairwise comparisons showed that the difference in HBO activation between the lecture teaching group (-.013 ± .005) and the control group (-.003 ± .005) was not significant (p =.14). The body rhythm teaching group (.012 ± .005) had significantly higher HBO activation than the control group (p =.034) and the lecture teaching group ($p < 0.001$). In ldlPFC activation, the main effect of teaching method was significant, $F(2, 100) = 7.08$, $p_{FDR} = .009$, $\eta_p^2 = 0.12$. Pairwise comparisons showed that the difference in HBO activation between the lecture teaching group (-.01 ± .006) and the control group (-.006 ± .006) was not significant (p =.627). The body rhythm teaching group (.017 ± .006) had significantly higher HBO activation than the control group (p =.004) and the lecture teaching group (p =.001), See Figure

S2c.

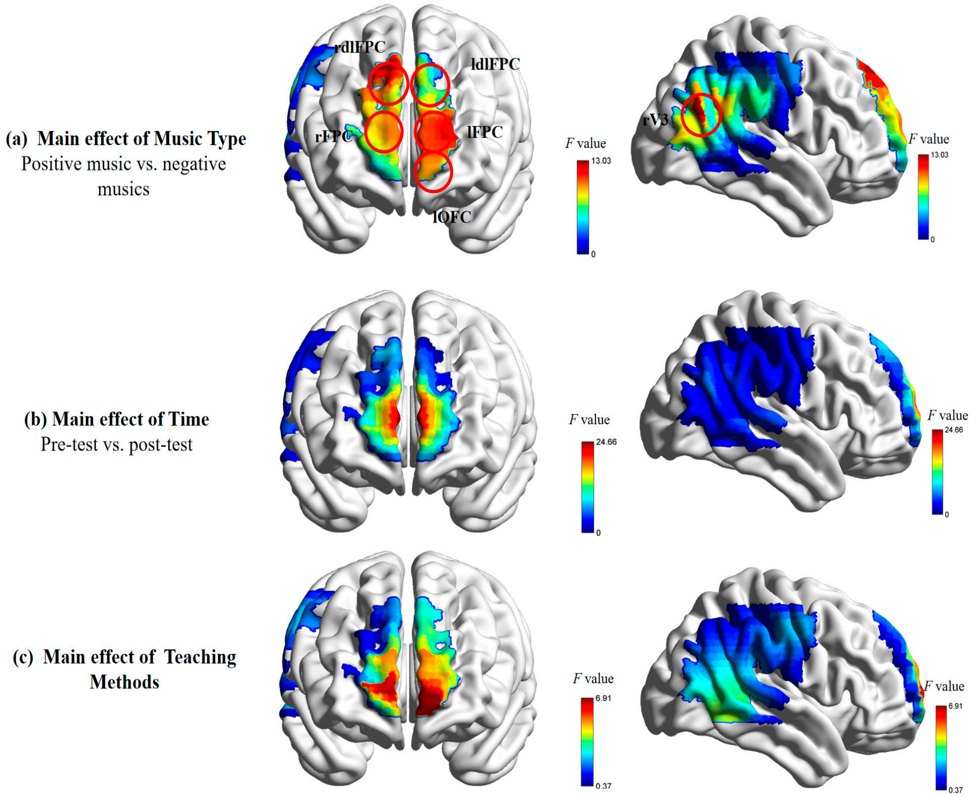

Figure S2. Results of analysis of variance. (a) Main effect of music type. (b) Main effect of time. (c) Main effect of teaching method.

S5.2.2 IBS Results Supplement

Table S6. ***Means and standard deviations of all channels of IBS during the teaching phase in the three groups（N=103）***

Variables	Lecture teaching group (n = 35)		Body rhythms teaching group（n = 35）		Control group （n = 33）	
	**Positive Music**	**Negative Music**	**Positive Music**	**Negative Music**	**Positive Music**	**Negative Music**
	M±*SD*	*M*±*SD*	*M*±*SD*	*M*±*SD*	*M*±*SD*	*M*±*SD*
lFPC	-0.034±0.012	-0.021±0.016	0.034±0.012	0.015±0.016	-0.006±0.012	-0.042±0.016
rFPC	-0.011±0.013	-0.011±0.017	0.021±0.013	0.011±0.017	0.01±0.013	-0.025±0.018

lOFC	-0.051±0.016	-0.033±0.019	0.051±0.016	0.029±0.019	-0.002±0.016	-0.028±0.019
rOFC	-0.011±0.019	-0.004±0.02	0.026±0.019	0.026±0.02	0.007±0.019	-0.039±0.021
ldlPFC	-0.005±0.014	0.001±0.016	0.03±0.014	0.005±0.016	0.002±0.014	0.002±0.016
rdlPFC	-0.005±0.017	0.009±0.019	0.013±0.017	0.004±0.019	-0.005±0.017	0.001±0.02
rSI	0.002±0.02	-0.001±0.021	0.039±0.02	0.045±0.021	-0.026±0.021	-0.021±0.021
rANG	-0.004±0.012	-0.002±0.013	0.011±0.012	0.017±0.013	-0.004±0.013	-0.0001±0.013
rSMG	-0.0003±0.014	-0.003±0.015	0.037±0.014	0.013±0.015	-0.002±0.015	-0.002±0.016
rITG	0.02±0.016	0.023±0.016	0.009±0.016	-0.005±0.016	0.052±0.017	0.041±0.017
rTPG	0.013±0.017	0.002±0.018	0.033±0.017	0.03±0.018	0.009±0.018	-0.026±0.018
rFFG	-0.025±0.018	-0.033±0.017	0.036±0.018	0.021±0.017	-0.014±0.018	-0.014±0.018
rV3	0.007±0.016	-0.016±0.016	0.04±0.016	0.028±0.016	0.004±0.016	0.001±0.016
rSTG	0.001±0.017	-0.007±0.018	0.032±0.017	0.042±0.018	-0.039±0.018	-0.025±0.019

A 3 (teaching method: lecture teaching group, body rhythm group, control group) × 2 (music valence: positive, negative) two-way repeated measures ANOVA with FDR correction for p-values for all channel results was performed on the mean IBS values for the three teaching phases, with the following complementary results: the IBS was significant in the lFPC (BA10, CH4 (F (2, 100) = 4.2, p_{FDR} = . 043, η_p^2 = .04) in which the main effect of music was significant. IBS activation by positive music in CH4 (0±0.09) was significantly higher than negative music (-.02±0.011; p = .043

S5.2.3. Behavior-brain correlation analysis

To examine the potential neural mechanisms underlying the behavioral advantages of the Body Rhythm Teaching Method, our correlation analysis directly focused on neurobehavioral associations within each teaching group. We hypothesized that specific, robust neurobehavioral coupling would emerge only in the Body Rhythm Teaching group—which demonstrated significantly superior emotional arousal scores compared to the other two groups (see Section 3.1). Accordingly, we calculated Pearson correlation coefficients between behavioral measures (residual change scores for emotional arousal) and brain activity measures (residual change scores for lFPC, rFPC, and IBSlOFC, IBSlFPC, IBSrSTG) across all three groups under both music conditions. The residual method was chosen to statistically control for baseline differences in pre-test scores across individuals, thereby isolating variation in post-test scores attributable solely to the experimental intervention (Cohen et al., 2013). All correlation coefficients underwent FDR correction to control

for multiple comparisons.

Correlation analysis revealed a highly condition-dependent and group-specific pattern of neurobehavioral coupling, providing empirical evidence for the distinct neural mechanisms underlying different teaching methods. Under positive music conditions, a significant behavior-brain association was observed exclusively in the body rhythm teaching group: after FDR correction, heightened emotional arousal showed a significant positive correlation with increased activity in the rFPC($r = 0.39$, $p_{FDR} = 0.03$). Additionally, we identified a trend that, while not reaching FDR-corrected significance, aligns with theoretical expectations: emotional arousal showed a marginally significant correlation with activity in the lFPC ($r = 0.33$, $p = 0.053$, $p_{FDR} = 0.068$). In stark contrast, no significant correlations were observed under identical conditions in either the lecture-based teaching group or the control group (all $p_{FDR} > 0.10$; Figure S3a). This pattern strongly suggests that the behavioral advantage of the body rhythm teaching method in positive musical contexts may stem from its ability to efficiently and synergistically mobilize bilateral prefrontal resources. The prefrontal cortex is typically involved in the cognitive regulation of emotions (Burgess et al., 2003) and is responsible for integrating attention allocation to external stimuli with the maintenance of internally generated cognitive processes (Kreplin & Fairclough, 2013). This study suggests that body rhythm teaching may more effectively guide learners toward deep cognitive integration and regulation of positive musical stimuli, thereby amplifying emotional responses—potentially representing the neural basis for its behavioral enhancement effects.

2

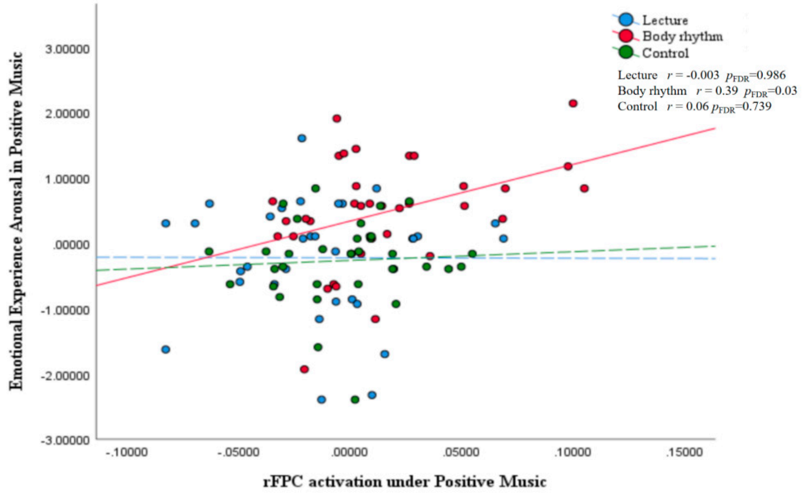

(a)

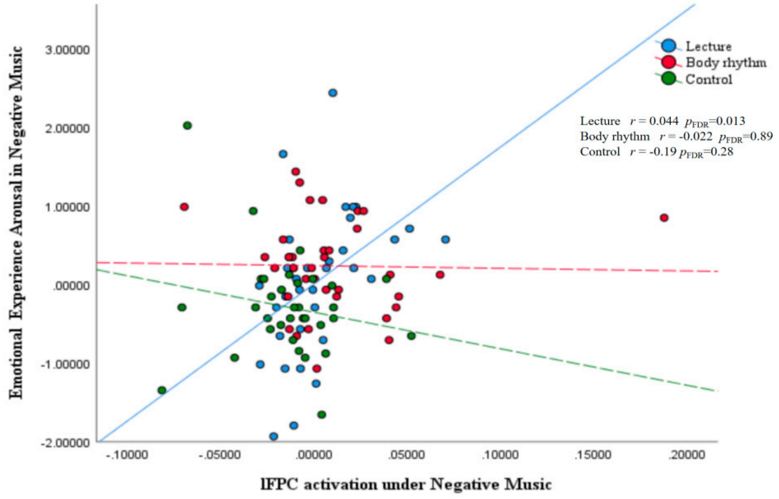

(b)

Figure S3. Scatter plots showing correlations between brain activation and emotional arousal levels. (a) Scatter plots and fitted curves for rFPC brain region activation versus emotional arousal changes under positive music conditions across different groups. The body rhythm teaching group (red data points and trend line) exhibited a significant positive correlation, while the lecture teaching group (blue) and Control group (green) showed no statistically significant correlations. (b) Scatter plot and fitted curve of lFPC brain region activation versus emotional arousal level changes under negative music conditions across different groups. The lecture teaching group (blue data points and trend line) showed a significant positive correlation, while

the body rhythm teaching group (red) and control group (green) showed no statistically significant correlation.

Additionally, under negative musical conditions, we observed a distinctly different pattern of neurobehavioral coupling. Only in the lecture teaching group did heightened emotional arousal show a significant positive correlation with lFPC activity ($r = 0.44$, $p_{FDR} = 0.013$; Figure S3b), whereas neither the body rhythm teaching group nor the control group exhibited such a significant association. This finding suggests that the lecture teaching method may engage distinct neurocognitive strategies for regulating emotional learning compared to body rhythm teaching method, with this mechanism being particularly pronounced when processing negative emotional content. Previous research indicates that the left prefrontal cortex is involved in cognitive processes such as language processing and deductive reasoning (Coetzee & Monti, 2018). We hypothesize that the lecture teaching method may prompt learners to engage in higher-order "analysis" and "semantic understanding" of negative emotional content, thereby specifically inducing lFPC-behavior coupling. This neural mechanism partially explains why the lecture teaching method outperformed the other two groups even under negative musical conditions.

In summary, this study reveals the multi-path neural-behavioral mechanisms through which different teaching methods influence musical emotion processing. The body rhythm teaching method exhibits an "enhancement-type" processing mechanism

characterized by bilateral FPC involvement under positive-emotion music conditions. Conversely, under negative-emotion conditions, the lecture teaching method demonstrates an “analytical-type” processing pathway dependent on the lFPC. The control group showed no organized neurobehavioral coupling patterns. Notably, although the lecture teaching method did not significantly outperform the control group in emotional arousal levels, it exhibited a specific and significant correlation between emotional arousal and lFPC activation under negative music conditions. This finding suggests that different teaching methods not only recruit distinct brain networks but also exhibit fundamental differences in the efficiency of their neural mechanisms. The neural resources relied upon by the body rhythm teaching method (such as bilateral FPC) can efficiently translate into behavioral-level emotional enhancement, thereby demonstrating comprehensive advantages in behavioral metrics. Conversely, while the lecture teaching method can mobilize lFPC, its neural activation fails to effectively translate into significant behavioral improvement, indicating a certain degree of “inefficiency” in this mechanism at the behavioral output level. Therefore, this study demonstrates that the neural mechanisms underlying the Body Rhythm Teaching Method exhibit high behavioral relevance and efficiency, whereas the mechanisms involved in the Lecture Teaching Method, though capable of specific activation, show limited efficacy in translating into behavioral outcomes. This finding provides empirical evidence for the superiority of the Body Rhythm Teaching Method at the neural mechanism level.

References

Burgess, P. W., Scott, S. K., and Frith, C. D. (2003). The role of the rostral frontal cortex (area 10) in prospective memory: a lateral versus medial dissociation. *Neuropsychologia* 41, 906–918. https://doi.org/10.1016/s0028-3932(02)00327-5

Cohen, J., Cohen, P., West, S. G., & Aiken, L. S. (2013). *Applied multiple regression/correlation analysis for the behavioral sciences*. Routledge.

Coetzee, J. P., & Monti, M. M. (2018). At the core of reasoning: Dissociating deductive and non-deductive load. *Human Brain Mapping, 39*(4), 1850-1861. https://doi.org/10.1002/hbm.23979

Chang, C., & Glover, G. H. (2010). Time – frequency dynamics of resting-state brain connectivity measured with fMRI. *Neuroimage, 50*(1), 81-98. https://doi.org/10.1016/j.neuroimage.2009.12.011

Cheng, X., Li, X., & Hu, Y. (2015). Synchronous brain activity during cooperative exchange depends on gender of partner: A fNIRS-based hyperscanning study. *Human Brain Mapping, 36*(6), 2039-2048. https://doi.org/10.1002/hbm.22754

Kreplin, U., & Fairclough, S. H. (2013). Activation of the rostromedial prefrontal cortex during the experience of positive emotion in the context of esthetic experience. An fNIRS study. *Frontiers in Human Neuroscience*, 7, 879. https://doi.org/10.3389/fnhum.2013.00879

Long, Y., Chen, C., Wu, K., Zhou, S., Zhou, F., Zheng, L., Zhao, H., Zhai, Y., & Lu, C. (2022). Interpersonal conflict increases interpersonal neural synchronization in

romantic couples. *Cerebral Cortex, 32*(15), 3254–3268. https://doi.org/10.1093/cercor/bhab413

Minako, U., Ippeita, D., Haruka, D., Yasushi, K., Taguchi, Y. H., & Eiju, W. (2015). Exploring effective multiplicity in multichannel functional near-infrared spectroscopy using eigenvalues of correlation matrices. *Neurophotonics, 2*(1), 1-7. https://doi.org/10.1117/1.nph.2.1.015002

Mutlu, M. C., Erdoğan, S. B., Öztürk, O. C., Canbeyli, R., & Saybaşılı, H. (2020). Functional near-infrared spectroscopy indicates that asymmetric right hemispheric activation in mental rotation of a jigsaw puzzle decreases with task difficulty. *Frontiers in Human Neuroscience*, 14, 252-252. https://doi.org/10.3389/fnhum.2020.00252

Pan, Y., Dikker, S., Goldstein, P., Zhu, Y., Yang, C., & Hu, Y. (2020). Instructor-learner brain coupling discriminates between instructional approaches and predicts learning. *NeuroImage,* 211, 116657. https://doi.org/10.1016/j.neuroimage.2020.116657

Schneider, P., Piper, S., Schmitz, C. H., Schreiter, N. F., Volkwein, N., Lüdemann, L., et al. (2011). Fast 3D near-infrared breast imaging using indocyanine green for detection and characterization of breast lesions. *Röfo*, 183, 956–963. https://doi.org/10.1055/s-0031-1281726

Torrence, C., Compo, G.P.(1998). A practical guide to wavelet analysis. Bull. Am. Meteorol.Soc. 79, 61 – 78.

Tong, Y., Lindsey, K. P., & deB Frederick, B. (2011). Partitioning of physiological noise signals in the brain with concurrent near-infrared spectroscopy and fMRI. *Journal of Cerebral Blood Flow & Metabolism, 31*(12), 2352-2362. https://doi.org/10.1038/jcbfm.2011.100

van Holstein, M., & Floresco, S. B. (2020). Dissociable roles for the ventral and dorsal medial prefrontal cortex in cue-guided risk/reward decision making. *Neuropsychopharmacology*, 45, 683-693. https://doi.org/10.1038/s41386-019-0557-7

Wang, S., Lu, J., Yu, M., Wang, X., & Shangguan, C. (2022). “I'm listening, did it make any difference to your negative emotions?” Evidence from hyperscanning. *Neuroscience Letters*, 788, 136865. https://doi.org/10.1016/j.neulet.2022.136865

Zheng, L., Liu, W., Long, Y., Zhai, Y., Zhao, H., Bai, X., ... & Lu, C. (2020). Affiliative bonding between teachers and students through interpersonal synchronisation in brain activity. *Social Cognitive and Affective Neuroscience, 15*(1), 97-109. https://doi.org/10.1093/scan/nsaa016

Zhou, S., Xu, X., He, X., Zhou, F., Zhai, Y., Chen, J., Long, Y., Zheng, L., & Lu, C. (2022). Biasing the neurocognitive processing of videos with the presence of a real cultural other. *Cerebral Cortex,* 1 – 14. https://doi.org/10.1093/cercor/bhac122

Zillekens, I. C., Schliephake, L. M., Brandi, M. L., & Schilbach, L. (2019). A look at actions: direct gaze modulates functional connectivity of the right TPJ with an

action control network. *Social cognitive and affective neuroscience, 14*(9), 977 – 986. https://doi.org/10.1093/scan/nsz07
